# New avenue of diagnostic stewardship: procedural stewardship for recurrent urinary tract infections in female patients

**DOI:** 10.1017/ash.2023.507

**Published:** 2023-12-12

**Authors:** Tanner D. Corse, Linda Dayan Rahmani, Hunter L. Hasley, Katherine Kim, Robert Harrison, Debra L. Fromer

**Affiliations:** 1 Department of Urology, Hackensack Meridian School of Medicine, Nutley, NJ, USA; 2 Department of Urology, Hackensack University Medical Center, Hackensack, NJ, USA

## Abstract

**Introduction:**

Societal guidelines offer a weak recommendation to perform cystoscopy for female patients with recurrent urinary tract infections (rUTI) of advanced age and/or with high-risk features. These guidelines lack the support of robust data and are instead based on expert opinion. In this retrospective cohort study, we aim to determine the utility of cystoscopy in patients with and without high-risk features for rUTI.

**Materials and methods:**

We identified 476 women who underwent cystoscopy for the evaluation of rUTI at a single tertiary academic medical center from May 1, 2015 and March 15, 2021. Patients were excluded if they had a competing indication for cystoscopy. Risk factors, demographic information, cystoscopic findings, and patient outcomes were analyzed.

**Results:**

192 (41.1%) were classified as having complicated UTI. We identified six patients (1.3%) with findings that prompted management to significantly impact patient outcomes. All six patients had high-risk features. 14 patients (3.0%) were found to have mucosal abnormalities prompting biopsy, three of which required general anesthesia. All 14 biopsies were ultimately benign.

**Conclusions:**

Our findings demonstrate a low diagnostic yield and increased risk exposure for women undergoing cystoscopy for the evaluation of complicated rUTI. Additionally, our observations support prior studies indicating that cystoscopy has limited utility in the evaluation of rUTI without high-risk features.

## Introduction

Urinary tract infections (UTI) are among the most common bacterial infections in women with a lifetime incidence of 50%–60%.^1–3^ Symptoms may involve urinary frequency, urgency, suprapubic discomfort, and dysuria, with potential complications including pyelonephritis and sepsis.^4^ Women with one UTI are at a higher risk of developing another, with 25%–50% of women experiencing at least one recurrent episode.^4–6^ Postmenopausal and young, sexually active women are at increased risk for recurrence with significant impacts on patient quality of life.^1,7–9^

Recurrent UTI (rUTI) is defined by both the 2019 joint American Urological Association/Canadian Urological Association/Society of Urodynamics, Female Pelvic Medicine & Urogenital Reconstruction (AUA/CUA/SUFU) and the 2022 European Association of Urology (EAU) guidelines as two separate, culture-proven episodes of acute bacterial cystitis with associated symptoms within six months or three episodes within one year.^10,11^ Included in both guidelines are classifications for uncomplicated and complicated UTI in female patients. UTI in male patients are significantly less common, considered complicated, and often present with prostate involvement.^4^ The AUA/CUA/SUFU guidelines define the index patient as an otherwise healthy adult female with an uncomplicated rUTI.^10^ The guidelines further describe uncomplicated rUTI as cases occurring in the index patient who has no known risk factors that would make her more susceptible to recurrence and complicated rUTI as cases involving a patient with high-risk features that may be put at increased risk for recurrence and/or decreased treatment efficacy.^10^ These high-risk features include anatomic or functional abnormalities of the urinary tract (eg, stone disease, diverticulum, neurogenic bladder), an immunocompromised host, or infection with multidrug-resistant (MDR) bacteria.^10^ The guidelines state that recommendations do not apply to women that are pregnant or exhibit signs of pyelonephritis, bacteremia, or other systemic infection. Furthermore, the American College of Radiology (ACR) Committee on Appropriate Use Criteria lists a total of 19 high-risk features that may deem a rUTI patient as complicated.^12^

**Figure 1.**
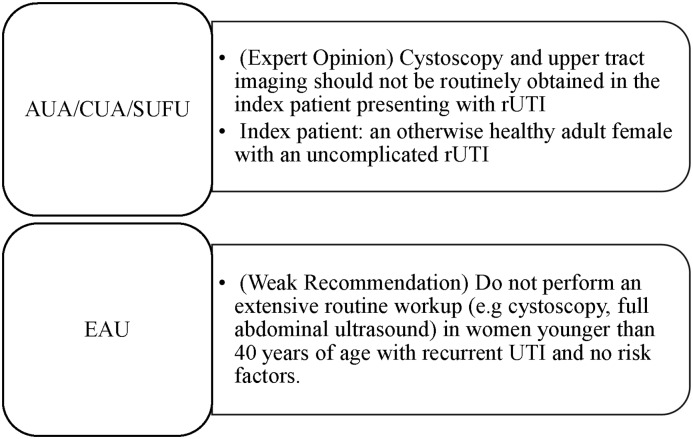
Societal recommendations regarding the use of cystoscopy for recurrent urinary tract infections.

The goal of cystoscopy in this patient population is to screen for anatomic abnormalities that may be causing persistent bacterial colonization within the urinary tract.^13–16^ The risks of complications from cystoscopy have been previously described, with discomfort and iatrogenic UTI (<2% incidence) being the most frequently reported.^17–19^ Recent studies examining women with rUTI undergoing cystoscopy by Pat et al. (*n* = 379), Dokubo et al. (*n* = 236), and Dieter et al. (*n* = 173) demonstrated diagnostic yields of 0.26%, 3.4%, and 2%, respectively, including findings such as calculi, diverticuli, fistulae, and bladder tumors.^20–22^ Urologic societies consistently recommend reserving cystoscopy for patients with high-risk features and/or of advanced age, although these recommendations are based upon expert opinion and lack robust supporting evidence.^10–12^ The EAU offers a “weak” recommendation to avoid cystoscopy in women without risk factors that are less than 40 years of age.^11^ A summary of cystoscopy guidelines across societies is shown in Figure 1. While there exists an increasing body of evidence to suggest that cystoscopy offers little to no clinical benefit in the evaluation and subsequent management of female patients with uncomplicated rUTI, there still remains a significant lack of evidence evaluating the utility of cystoscopy in complicated rUTI patients or those of advanced age.^10–12,20–25^ A recent “Guideline of Guidelines” on rUTI found most recommendations aimed at otherwise healthy non-pregnant women with uncomplicated cystitis and suggested further recommendations to assist management of complex patient groups, such as patients with complicated rUTI.^26^

This retrospective cohort study seeks to build an evidentiary basis for the utility of cystoscopy in the evaluation of patients with rUTI by analyzing a larger cohort with a more robust classification of high-risk features than previous studies.

## Methods

**Figure 2.**
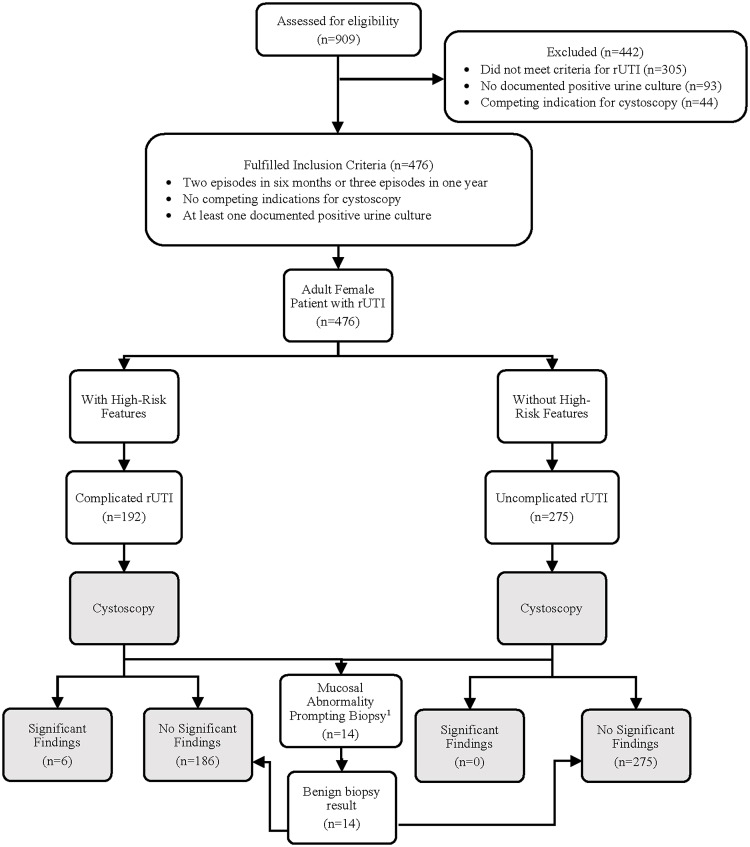
Flowchart breakdown of patients with recurrent urinary tract infections to results.^1^Mucosal abnormalities prompted biopsies in 14 patients, of which all returned benign results with no significant findings.

Billing codes were used in this institutional review board (IRB) approved study (Pro2020-0946) to retrospectively identify 909 females that underwent cystoscopy between May 1, 2015 and March 15, 2021 at a single tertiary academic medical center. Patients were included if they had two documented symptomatic UTIs within six months or three within one year with at least one documented positive urine culture (>10^5^ colony-forming units/mL). Patients only met criteria of an episode of UTI with positive urine culture if they also experienced symptoms of UTI. Patients were excluded if they did not meet criteria for rUTI, were less than 18 years of age, had a competing indication for cystoscopy (eg, known malignancy, gross hematuria in the absence of current UTI, or known anatomic abnormalities), or did not have a documented urine culture (Figure 2). Patient data were accessed through the hospital’s online medical record system, EPIC® (Verona, Wisconsin, Epic Systems Corporation), and stored in a database constructed via REDCap.^27,28^ Patients did not routinely receive prophylactic antibiotics following screening cystoscopy.

Using a combination of the high-risk features outlined in the 2019 AUA/CUA/SUFU guideline, 2017 EAU guideline, and the criteria set forth by the ACR committee, we classified UTIs as complicated if patients had any of the following high-risk features: urolithiasis, diverticula, neurogenic bladder, prior urogynecological surgery (not including hysterectomy and cesarean section), pregnancy, immunocompromising conditions, positive urine culture for MDR bacteria, bacteremia, pyelonephritis, and/or lack of appropriate response to treatment of uncomplicated UTI including suppressive antibiotics.^10–12^ MDR organisms were defined as those with non-susceptibility to at least one agent in three or more antimicrobial categories.^29^ The two comparative groups for this retrospective cohort study include patients with high-risk features and those without high-risk features, as shown in Figure 2. Changes in management due to findings on cystoscopy were defined as incidents where a patient initially treated using the standard of care of conservative therapy (eg, behavioral changes, topical estrogen, D-mannose) and/or continuous low-dose antibiotic suppression was subsequently evaluated or where treatment escalated such as with invasive and/or surgical procedures with or without the requirement for anesthesia.^30–34^

Demographic information, high-risk features for complicated UTI, cystoscopic findings, and outcomes of medical and surgical management were collected. Descriptive statistics including measures of central tendency (medians) and measures of variability (interquartile ranges (IQR)) were performed to analyze patient demographics involved in baseline characteristics and significant cystoscopic findings. Ratios of values were used for categorical variables. The number of patients needed to undergo cystoscopy to identify a positive finding was based on formula for number needed to treat, with the number of patients needed to be treated to prevent an adverse event.

## Results

Demographic characteristics are summarized in Table 1. We identified 467 women who underwent cystoscopy for rUTI. The median age was 64 years (IQR 51–74). The median BMI was 26.2 (SD = 6.6). In terms of race, 336 (70.6%) self-identified as White, 26 (5.5%) as Asian, nine (1.9%) as African American, one (0.21%) as Native American, eight (1.7%) as more than one race, 22 (4.6%) did not report their race, and 65 (13.7%) identified as Other. 192 (41.1%) patients were classified as having complicated rUTI based on the presence of one or more high-risk features. We identified a total of 221 high-risk features (Table 1), of which there were 54 patients with prior urogynecological surgery (24.4%), 39 with a history of pyelonephritis, fevers, or bacteremia (17.7%), 39 cases of culture-proven MDR organisms (24.4%), 21 cases of active nephrolithiasis (9.9%), 19 patients with neurogenic bladder and urinary retention secondary to diabetes mellitus, spinal cord injury, stroke, or urinary retention (8.6%), and 14 immunocompromised patients (6.3%) including both those patients on immunomodulators and patients suffering from a specific immunodeficiency.

**Table 1.** Baseline characteristics of female patients undergoing cystoscopy for rUTI

Variables	Count (*N* = 476)
Age, years, median (IQR)	64 (51–74)
Body mass index (BMI), kg/m^2^, median (IQR)	26.2 (23.3–30.3)
Race (%)	
White	336 (70.6%)
Asian	26 (5.5%)
African American	9 (1.9%)
Native Hawaiian or Other Pacific Islander	1 (0.21%)
More than one race	8 (1.7%)
Unknown/Not reported	22 (4.6%)
Other	65 (13.7%)
High-risk features (%)	
Prior urogynecological surgery	54 (24.4%)
Pyelonephritis/Fevers/Bacteremia	39 (17.6%)
MDR bacteria	39 (17.6%)
Nephrolithiasis	21 (9.5%)
Neurogenic bladder	19 (8.6%)
Immunocompromised	14 (6.3%)
Chemotherapy	8 (3.6%)
Lack of appropriate response to therapy	8 (3.6%)
Catheter use	8 (3.6%)
Post-transplantation	5 (2.3%)
Pregnancy	1 (0.45%)
Other	5 (2.3%)

Significant findings on cystoscopy are summarized in Table 2. In total, six patients were identified with significant cystoscopic findings that altered subsequent management: bladder stones in patients with neurogenic bladder (2), high-grade non-invasive bladder cancer (1), lymphoma (1), urethral erosion of mesh sling (1), and urachal remnant (1). All 6 patients had high-risk features that fulfilled the criteria for complicated rUTI (Table 2). The median age of this group was 58 years (range: 44–71). A total of 14 (3.0%) patients classified in both complicated and uncomplicated rUTI groups (Figure 2) were found to have mucosal abnormalities that prompted cystoscopic biopsy, three of which required general anesthesia. Of these 14 cases, all were benign including findings of cystitis glandularis (1) and papilloma (1). Further biopsy and general anesthesia were the only additional recorded complications from cystoscopy.

**Table 2.** Summary of significant cystoscopic findings in female patients with recurrent urinary tract infections

Variables	Count (*N* = 6)
Age, years, median (IQR)	58.0 (44–71)
Findings (%)	
Bladder stone	2 (33.3%)
Bladder cancer^a^	1 (16.7%)
Lymphoma	1 (16.7%)
Urethral erosion of mesh sling	1 (16.7%)
Urachal remnant	1 (16.7%)

a High-grade, non-invasive.

## Discussion

To the best of our knowledge, our study represents the largest cohort to date examining the cystoscopic findings of female patients with rUTI. Major urologic societal guidelines regarding the utility of cystoscopy in this patient population currently lack robust data and are based solely on expert opinion, particularly for patient populations with high-risk features that fulfill the criteria for complicated rUTI. Our analysis supports prior observations that the diagnostic yield of cystoscopy in the index rUTI patient population is not only limited, but that the diagnostic yield in patients of advanced age and with complicated rUTI is minimal as well. Additionally, the study demonstrates that cystoscopy is not without risk as clinically insignificant findings can lead to further evaluation with increased risk exposures.

The diagnostic yield of cystoscopy in female patients with rUTI in the literature varies from 0 to 7.6%, with cohort sizes ranging from 15 to 379.^20–25^ Only 1.3% (6/467) of cystoscopies in this cohort demonstrated significant findings that altered management. It is important to point out that significant findings among all patients were exclusively in the cohort of patients with high-risk features/complicated rUTI. Though all six of these patients fulfilled the criteria for complicated rUTI, these six represented only 3.1% (6/192) of all patients with complicated rUTI (Table 2). The complicated rUTI patients would be the only group undergoing cystoscopy had current guidelines been strictly adhered to. There were no significant findings on cystoscopy among patients with uncomplicated rUTI.

Prior studies excluded certain high-risk features which can translate to lower diagnostic yields. Pagano et al. excluded patients with hematuria in the presence of UTI.^24^ Despite our limited exclusions, our combined rate is lower (1.3%, 6/467) than that of Pagano et al (3.8%).^24^ While two life-threatening conditions were identified on cystoscopy (both carcinoma), these findings made up just 0.42% of all patients (2/476) and 1.0% of complicated rUTI patients (2/192). This is consistent with the rate of life-threatening cystoscopic findings in rUTI patients by Santoni et al. and Pat et al. of 0.15% and 0.26%, respectively.^20,25^ There is no clear consensus as to the threshold for a positive yield that should warrant cystoscopy, although a yield of at least 5% has been previously suggested.^25^ In our study, the number of patients with complicated rUTI with positive findings was 6 out of 192 patients (3.1%). In other words, the number of patients with complicated rUTI needed to undergo cystoscopy to identify one positive finding was 32, demonstrating relatively low yield.

It is important to note that the criteria regarding which high-risk factors qualify a rUTI patient as “complicated” varies between medical societies. We included patients that fit all three criteria from the joint 2019 AUA/SUFU/CUA guideline: (1) history of anatomic or functional abnormalities of the urinary tract, (2) immunocompromising conditions, and (3) MDR organisms. Patients with signs of pyelonephritis and other systemic infections were also included in our analysis, in line with the ACR’s criteria.^12^ We also included all pregnant patients in accordance with the 2022 EAU Guideline.^11^ Of the 19 high-risk features detailed by the ACR, there are six that we did not consider in our criteria for complicated rUTI (elevated serum creatinine, asymptomatic bacteriuria, severe diabetes mellitus, childhood UTI, analgesic abuse, and urinary incontinence) as we felt they did not align with AUA/CUA/SUFU and EAU guidelines.^10–12^

While the EAU guideline recommends against the use of cystoscopy in patients without high-risk features under 40 years of age, they do not offer recommendations for the management of patients of advanced age.^11^ Notably, the median age of our cohort (64.0 years) is over two decades older than the EAU’s cutoff.^11^ The median age of patients with high-risk features and significant findings on cystoscopy was 58 years. These findings suggest that the EAU guideline’s recommendation for all patients with high-risk features to undergo cystoscopic evaluation regardless of age may be of limited clinical benefit, and the cutoff age recommending against cystoscopy may have the potential to be increased.^11^ Our diagnostic yield for patients over the age of 55 years old is consistent with the 3.4% yield demonstrated by Dokubo et al.^21^ Our findings support the AUA’s expert opinion guideline of discouraging cystoscopy in the index patient, but importantly suggest that the AUA/CUA/SUFU’s, ACR’s, and EAU’s recommendations to consider cystoscopy in patients of advanced age or with high-risk features may be of low diagnostic yield.^10–12^ Future work may also focus on patient menopausal status in addition to age. Further stratification of patients by risk factors including sexual activity or use of spermicides may also be of clinical significance.^1,4^

In addition to the low yield for cystoscopy in this patient population, we demonstrated an increased exposure to risks via continued diagnostic workup prompted by findings on cystoscopy. The risks of complications from cystoscopy, including discomfort and post-procedural UTI, have been previously described.^17–19^ However, we demonstrated escalating diagnostic evaluation of three patients that necessitated general anesthesia to tolerate mucosal biopsy. All three of these patients ultimately yielded benign biopsy findings. This highlights the need for shared decision-making between providers and this patient population when weighing the benefits and risks of undergoing cystoscopy for the evaluation of rUTI. A recent systematic review of existing clinical practice guidelines for the assessment and treatment of rUTI found that only five of the eight identified guidelines took patients’ perspectives into account when developing their recommendations and none of the guidelines publicly reported those perspectives.^35^ Additionally, optional imaging demonstrates a significant cost burden to this patient population with Gaitonde et al. calculating the cost of initial evaluation for patients with rUTI being $730 and $390 with and without optional imaging, respectively.^36^ Patients must be informed of the diagnostic yield in the face of the potential risks and costs of pursuing a diagnostic workup in order to achieve appropriate shared decision-making.

Our study is subject to several limitations. It was conducted at a single center in a retrospective manner with heterogeneity among patients regarding prior treatments and management among several treating physicians, with updated guidelines and treatments throughout the observed period of study. Timeframe from first UTI to cystoscopy and types and duration of initial management, including but not limited to vaginal estrogen, were not analyzed in this study. Possible adverse events after diagnostic cystoscopy were also outside the scope of this study and should be examined as a point of further work. Additionally, the criteria for high-risk features for patients with rUTI vary among societal recommendations, and our criteria attempted to combine several guidelines in order to conduct our analysis. We did not correlate imaging data to cystoscopy findings to compare their utilities; however, of the six significant cystoscopic findings documented in our cohort, none would have warranted cystoscopy if preoperative imaging had been initially performed. Another limitation is that although we deemed cystitis cystica as a benign finding, recent preclinical and retrospective studies have suggested it may pose a risk factor in developing rUTIs.^37,38^ Certain research has also identified inflammatory lesions as a possible bacterial reservoir managed with electrofulguration; however, the identification of these lesions and further management was not included as significant findings in our study.^39,40^ The literature demonstrates a higher yield of cystoscopic findings in male rUTI patients, and so the findings in our female-only cohort should not be generalized to men.^10–12,21^

Prospective, randomized studies evaluating the correlation of high-risk features to the probability of obtaining significant cystoscopic findings are needed, but due to the relatively low diagnostic yield, they will require extremely large sample sizes in order to demonstrate statistical significance. Additionally, patients may request additional evaluation after extensive antibiotics or persistent organisms in subsequent cultures. Further research could examine patient satisfaction or patient-recorded outcomes and whether patients request imaging measures for reassurance.

Societal guidelines recommend that cystoscopy should not be obtained in the index rUTI patient, but do not specify their utility in patients >40 years and/or with high-risk features for complicated rUTI and/or urothelial malignancy. These recommendations are based on expert opinion and lack the support of robust evidence. In the largest retrospective study of female rUTI patients undergoing cystoscopy to date, cystoscopy demonstrated no clinical benefit and a low diagnostic yield in patients with uncomplicated and complicated rUTI, respectively. Our findings suggest that cystoscopy may have limited benefit with an increased risk exposure in complicated rUTI patients over the age of 50 and support the recommendation to avoid cystoscopy for rUTI patients without risk factors. Physicians should hold patient-oriented discussions with complicated rUTI patients regarding the likely yield of clinical benefit and risk exposure if considering further cystoscopic management. Further research regarding the utility of cystoscopy for patients with rUTI is needed with increasingly robust methodologic design.
